# ‘MicroRNA Targets’, a new AthaMap web-tool for genome-wide identification of miRNA targets in *Arabidopsis thaliana*

**DOI:** 10.1186/1756-0381-5-7

**Published:** 2012-07-16

**Authors:** Lorenz Bülow, Julio C Bolívar, Jonas Ruhe, Yuri Brill, Reinhard Hehl

**Affiliations:** 1 Institut für Genetik, Technische Universität Braunschweig, Spielmannstr, 38106, Braunschweig, Germany

**Keywords:** *Arabidopsis thaliana*, AthaMap, MicroRNAs, Small RNAs, Post-transcriptional regulation

## Abstract

**Background:**

The AthaMap database generates a genome-wide map for putative transcription factor binding sites for *A. thaliana*. When analyzing transcriptional regulation using AthaMap it may be important to learn which genes are also post-transcriptionally regulated by inhibitory RNAs. Therefore, a unified database for transcriptional and post-transcriptional regulation will be highly useful for the analysis of gene expression regulation.

**Methods:**

To identify putative microRNA target sites in the genome of *A. thaliana*, processed mature miRNAs from 243 annotated miRNA genes were used for screening with the psRNATarget web server. Positional information, target genes and the psRNATarget score for each target site were annotated to the AthaMap database. Furthermore, putative target sites for small RNAs from seven small RNA transcriptome datasets were used to determine small RNA target sites within the *A. thaliana* genome.

**Results:**

Putative 41,965 genome wide miRNA target sites and 10,442 miRNA target genes were identified in the *A. thaliana* genome. Taken together with genes targeted by small RNAs from small RNA transcriptome datasets, a total of 16,600 *A. thaliana* genes are putatively regulated by inhibitory RNAs. A novel web-tool, ‘MicroRNA Targets’, was integrated into AthaMap which permits the identification of genes predicted to be regulated by selected miRNAs. The predicted target genes are displayed with positional information and the psRNATarget score of the target site. Furthermore, putative target sites of small RNAs from selected tissue datasets can be identified with the new ‘Small RNA Targets’ web-tool.

**Conclusions:**

The integration of predicted miRNA and small RNA target sites with transcription factor binding sites will be useful for AthaMap-assisted gene expression analysis. URL: http://www.athamap.de/

## Background

Small RNAs play an important role in post-transcriptional gene expression regulation. Currently, five classes of small RNAs are described in plants. Heterochromatin associated siRNAs, *trans*-acting siRNAs, repeat associated siRNAs, naturally occurring antisense siRNAs, and miRNAs [1]. These small RNAs function as inhibitory RNAs (RNAi) by causing degradation of target mRNAs, inhibition of mRNA translation, or by eliciting epigenetic effects such as DNA methylation. The different classes of small RNAs are generated by distinct, sometimes converging pathways involving, among others, several DICER-LIKE proteins for processing double stranded RNA and several ARGONAUTE proteins for incorporating small RNAs into RNA induced silencing complexes (RISC) or RNA induced transcriptional silencing (RITS) complexes [2].

The fact that small RNAs have a high degree of complementarity to their target sites led to the establishment of bioinformatic resources for determining target sites and target genes. Towards these ends the miRBase database was established [3]. Initially, miRBase served as a species independent repository for miRNAs containing mainly structural data. Later on, predicted gene targets and expression data derived from RNA deep sequencing experiments were incorporated [4,5]. Currently, miRBase contains data for 243 different *A. thaliana* miRNA genes (miRBase, Release 16). In addition to miRBase, several other *A. thaliana* or plant specific databases with information on siRNAs and miRNAs are available such as ASRP, CSRDB, PMRD, and SoMART [6-10].

A major challenge for the bioinformatic prediction of miRNA regulated genes is the lack of identity between the miRNAs and their target sequences [11]. Several tools were developed for computer-assisted plant miRNA target prediction such as miRU and Tapir [12,13]. Based on experimentally verified target genes, Target-align and psRNATarget were developed for plant miRNA target detection [14,15]. These tools take into account the sequence identity as well as the positional conservation of mismatches between miRNAs and their target genes [16,17]. Furthermore, they consider recent findings on non-perfectly binding miRNAs and those leading to translational inhibition of the target mRNA [18,19].

The identification of genes targeted by inhibitory RNA is useful when studying gene expression regulation using databases on *cis*-regulatory elements or transcription factor binding sites such as AGRIS or AthaMap [20,21]. With these databases it is possible to predict regulatory sequences in gene promoters. Knowledge about the putative post-transcriptional regulation of the genes of interest will be important when studying gene expression regulation.

The work presented here describes the genome wide identification of miRNA target sites with the psRNATarget web server in *A. thaliana*. Based on genomic positions and orientation, putative target genes were identified. The genomic positions of miRNA target sites were integrated into AthaMap, a database for predicted transcription factor binding sites in the *A. thaliana* genome [22]. New web-tools were developed for the online identification of putative miRNA and small RNA target genes.

## Methods

### Annotation of miRNA target sites in AthaMap

For genome-wide miRNA target identification in *A. thaliana*, the psRNATarget web server at http://plantgrn.noble.org/psRNATarget/ was used online [15]. The psRNATarget web server contains 243 published miRNAs from the mirRBase database (Release 16, September 2010, [5]). Since transcripts from different miRNA genes can be processed into the same mature miRNA, target sites for closely related miRNA genes were identified with the same screening sequence. A total of 190 different screening sequences represent the 243 microRNA genes. To obtain positional information on putative miRNA target sites, the genomic sequence of *A. thaliana* (TAIR8) was fragmented into overlapping pieces to accommodate the size limitation of the web server. The following parameters were chosen online. The maximum expectation was set to 5.0 and flanking length around target site for target accessibility analysis was set to zero. All other parameters were left unchanged. After obtaining positional information of putative binding sites with corresponding psRNATarget score (range 0.0-5.0), the data was downloaded, and the fragmented *Arabidopsis* chromosomes were reassembled to obtain absolute positional information. This information was then integrated into the AthaMap database [23].

### AthaMap update with small RNA transcriptome datasets

In an earlier study, two small RNA transcriptome datasets from inflorescence and seedling tissue were used for the identification of small RNA target sites with the TAIR7 genome release [23,24]. Now, additional seven small RNA transcriptome datasets were used and target sites for all nine datasets were determined with the TAIR8 genome release [25]. GSM65747 and GSM65750 belong to the dataset collectively found under Gene Expression Omnibus (GEO) accession number GSE3008 [24]. GSM118372, GSM118373, GSM118374, and GSM118375 belong to the dataset collectively found under GEO accession number GSE5228 [26,27]. GSM154336, GSM154370, and GSM154375 belong to the dataset collectively found under GEO accession number GSE6682 [28-30]. These datasets contain between 8,112 and 141,539 individual sequences (Table 1). Datasets were downloaded from GEO at http://www.ncbi.nlm.nih.gov/geo[31] and the genomic positions of all small RNA sequences were determined. Towards these ends, the same Perl script was applied which was previously used for small RNA target site determination [23].

**Table 1:** Small RNA datasets used in the analysis

**GEO Accession**	**Dataset**	**Tissue**	**Number of sequences**	**Number of genomic hits**
GSE3008	GSM65747	inflorescence	67,528	243,803
	GSM65750	seedlings	42,062	159,304
GSE5228	GSM118372	flower	100,658	342,502
	GSM118373	leaf	67,663	307,180
	GSM118374	seedlings	77,937	332,590
	GSM118375	silique	141,539	470,537
GSE6682	GSM154336	inflorescence	57,966	170,621
	GSM154370	leaf	8,112	36,084
	GSM154375	seedlings	12,718	50,962

## Results and Discussion

### New web-tools for genome-wide identification of miRNA and small RNA targets

The genomic screens with the processed mature miRNAs determined between 121 and 314 putative target sites for each sequence. A total of 41,965 target sites were determined for all screening sequences in the genome of *A. thaliana*. Next, the number of genes putatively regulated by miRNAs was predicted. Therefore, target sites in all annotated genes were determined. A target site is defined as the reverse complement of the small RNAs in the annotated transcript. A total of 15,390 miRNA target sites were detected in *A. thaliana* genes within the transcript of the genes (Additional file 1, S1.xls). Because the same gene can have target sites for more than one miRNA, the total number of different genes was determined to be 10,442 (Additional file 2, S2.txt). For a further resolution of specific miRNA target sites, additional file 1 provides the gene ID of the transcript, the miRNA that may target this gene, the absolute chromosomal position of the miRNA target site (the chromosome is identified by the gene ID), and the maximum expectation (score) which was obtained for this target site with the psRNATarget web server.

**Figure 1:**
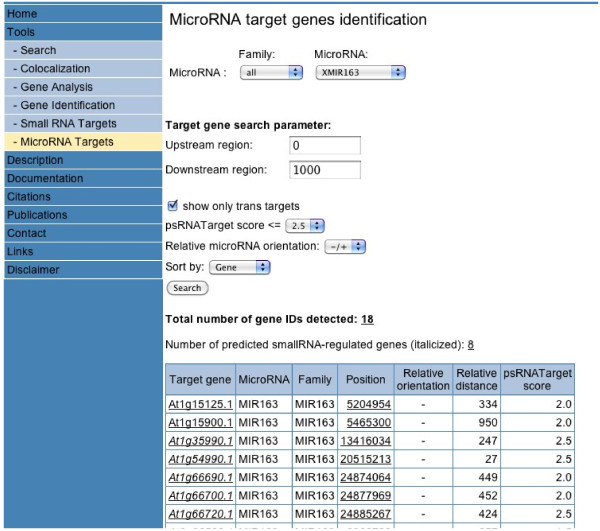
The ‘MicroRNA Targets’ web-tool after performing a search for miRNA163 target genes.

**Figure 2:**
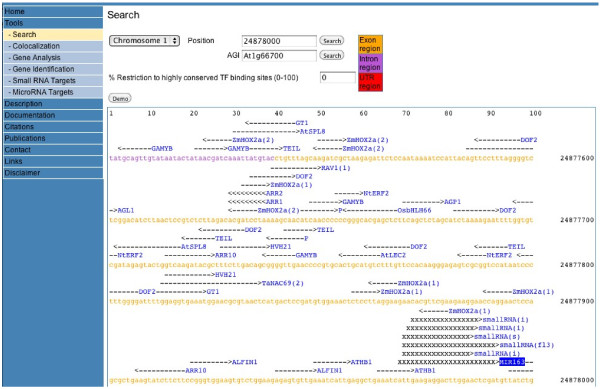
Partial screenshot of the sequence window linked to the miRNA163 target site at position 24877969 on chromosome 1.

To permit the identification of putative miRNA targets, the new web-tool ‘MicroRNA Targets’ was developed: http://www.athamap.de/miRNA_ident.php. After selecting the miRNA, user selected parameters are for example the sequence window to be analysed for each gene and the psRNATarget score (0.0-5.0). If a lower false positive prediction rate is preferred, a more stringent cut-off threshold (0.0-2.0) should be set. If a higher prediction coverage is desired, a more relaxed cut-off threshold (4.0-5.0) can be chosen. 0.0 means identity between miRNA and target site. Figure 1 shows a screen shot of this new tool with part of the result table obtained for a miRNA163 target site search with target search parameters. All target genes and the position of the target sites in these genes are linked to a sequence display window. Figure 2 shows a partial sequence display window for position 24877969 of target gene At1g66700.1. This not only displays the miRNA selected and the miRNA target site within the genomic sequence, but also shows that small RNAs were identified in different small RNA transcriptome datasets targeting the same chromosomal position. The number of genomic hits obtained for each small RNA transcriptome dataset ranges between 36,084 and 470,537 (Table 1). With dataset GSE3008, 5,929 genes were identified to harbour target sites of small RNAs (Additional file 3, S3.txt). In a similar way, 7,696 and 4,032 target genes have been determined for datasets GSE5228 and GSE6682, respectively (Additional files 4 and 5; S4.txt, S5.txt). 3,325 genes are common to all datasets (Additional file 6, S6.txt). In total, 9,166 different genes were predicted to be the target of a small RNA (Additional file 7, S7.txt). When target genes for miRNAs and small RNAs are taken together, 16,600 different genes are predicted to be the target of inhibitory small RNAs (Additional file 8, S8.txt).

In addition to the ‘MicroRNA Targets’ web-tool, putative small RNA targets can be predicted using a second novel web-tool called ‘Small RNA Targets’ accessible at http://www.athamap.de/smallRNA_targets.php. It permits identification of small RNA target genes for selected small RNA transcriptome datasets similar to the ‘MicroRNA Targets’ tool.

### Update of other AthaMap web-tools to identify putative post-transcriptionally regulated genes

Genes targeted by small RNAs and miRNAs are now identified in AthaMap at http://www.athamap.de when the web-tools ‘Colocalization’, ‘Gene-Analysis’, and ‘Gene Identification’ are used. With these web-tools one can determine, for example, putative combinatorial transcription factor binding sites (TFBS), common TFBS in a set of user submitted genes, or genome wide TFBS for user selected TFs [20,32,33]. On the result pages the number of putatively post-transcriptionally regulated genes targeted by small RNAs and/or miRNAs are identified and those genes are tagged with a gene ID in italics (small RNA), in bold (miRNA), or in italics and bold in the result table. Furthermore, putatively post-transcriptionally regulated genes can be omitted from the analysis by checking a box designated ‘exclude genes putatively regulated by small RNA’ and/or ‘exclude genes putatively regulated by miRNAs’. Furthermore, the ‘Gene Analysis’ web-tool which permits the graphical display of selected TFBS and small RNA target sites was complemented with an option to select miRNA target sites (MIR). When this option is selected, a graphic display of all submitted genes showing target sites of miRNAs within the selected gene region will be shown.

### Database assisted analysis of transcriptionally and post-transcriptionally regulated genes

The information on putative post-transcriptionally regulated genes may be valuable when determining target genes of transcription factors or transcription factor binding sites within the *A. thaliana* genome. This will add another level of specificity to the analysis because it permits the identification of genes potentially regulated by miRNAs and this regulation may complement the regulation by TFs. The expression of microRNA genes themselves is also tightly regulated [34,35]. Therefore, the tissue-specific or stress-specific induction or repression of a miRNA gene may downregulate or activate a target gene independently of TFs that target the same gene. Therefore, the integration of genes possibly targeted by small RNAs and/or miRNAs in a database for transcriptional regulation may contribute significantly to the functional analysis of gene expression regulation.

There are several *A. thaliana* specific databases that identify *cis*-regulatory sequences. For example AtcisDB which is part of the Arabidopsis Gene Regulatory Information Server (AGRIS) harbours experimentally verified and predicted *cis*-regulatory sequences in the upstream region of approximately 33,000 *A. thaliana* genes [21,36-38]. Another database, Athena, contains 30,067 predicted *Arabidopsis* promoter sequences and consensus sequences for 105 previously characterized transcription factor (TF) binding sites [39]. Also ATTED-II and PlantPan identify *cis*-regulatory sequences in *A. thaliana* genes [38,40-42]. In contrast to these databases, AthaMap, a database for transcription factor bindings sites for the whole *A. thaliana* genome, integrates transcriptional and post-transcriptional data [23]. The present work has extended the data content from 5,772 putatively post-transcriptionally regulated genes targeted by small RNAs to a total of 9,166 genes identified with small RNA transcriptome datasets. Most importantly, 10,442 putative target genes of mature miRNAs corresponding to 243 different microRNA genes were also identified. By tagging these genes in the AthaMap ‘Colocalization’, ‘Gene-Analysis’, and ‘Gene Identification’ web-tool results, the user can identify all putatively post-transcriptionally regulated genes and can also omit those genes in the analysis. Furthermore, the new web-tool ‘MicroRNA Targets’ reported here permits the identification of miRNA target genes in AthaMap. The identification and annotation of small RNA target sites also for intergenic regions may contribute to the functional analysis of small RNAs in epigenetic regulation of gene expression.

## Conclusions

The identification of genes targeted by inhibitory RNAs is useful when studying gene expression regulation using databases. With AthaMap it is possible to predict regulatory sequences in gene promoters and to identify those genes that are potentially targeted by inhibitory RNAs. With the annotation of putative miRNA target sites from processed mature miRNAs from 243 miRNA genes and small RNA target sites from nine small RNA transcriptome datasets a total of 16,600 *A. thaliana* genes are predicted to be potentially regulated by inhibitory RNAs.

## Competing Interests

The authors declare that they have no competing interests.

## Authors’ Contributions

LB and RH designed the work and wrote the paper. LB, JCB, and JR performed the genome wide target site screens with miRNAs and small RNA transcriptome data and annotated the data to AthaMap. YB programmed the web interface. All authors read and approved the final manuscript.

## Supplementary Material

Additional file 1 Positional information and score of target sites of miRNAs determined with psRNATarget in genes harbouring miRNA target sites within their transcripts. Click here for file

Additional file 2 A list of 10,442 different genes harbouring a target site for a miRNA detected with psRNATarget within their transcripts. Click here for file

Additional file 3 A list of 5,929 genes identified to harbour target sites of small RNAs with dataset GSE3008. Click here for file

Additional file 4 A list of 7,696 genes identified to harbour target sites of small RNAs with dataset GSE5228. Click here for file

Additional file 5 A list of 4,032 genes identified to harbour target sites of small RNAs with dataset GSE6682. Click here for file

Additional file 6 A list of 3,325 genes identified to harbour target sites of small RNAs with datasets GSE3008, GSE5228, and GSE6682 and common to all datasets. Click here for file

Additional file 7 A list of 9,166 all genes identified to harbour target sites of small RNAs with datasets GSE3008, GSE5228, and GSE6682. Click here for file

Additional file 8 A list of 16,600 genes identified to harbour target sites of small RNAs with datasets GSE3008, GSE5228, and GSE6682 and with miRNAs. Click here for file
